# Case report: when two and ½ men go camping…

**DOI:** 10.1186/s12879-017-2213-3

**Published:** 2017-01-30

**Authors:** Matthias von Rotz, Alexa Dierig, Ulrich Heininger, Carl Chrobak, Veronika Baettig, Adrian Egli

**Affiliations:** 1 Division of Infectious Diseases and Hospital Epidemiology, University Hospital Basel, Basel, Switzerland; 2 Department of Internal Medicine, University Hospital Basel, Basel, Switzerland; 3 Division of Pediatric Infectious Diseases, Children University Hospital Basel, Basel, Switzerland; 4 Applied Microbiology Research, Department of Biomedicine, University of Basel, Basel, Switzerland; 5 Clinical Microbiology, University Hospital Basel, Basel, Switzerland

**Keywords:** Listeria monocytogenes, Sepsis, MALDI-TOF, Rapid identification, Typing, Pulsed field gel electrophoresis

## Abstract

**Background:**

In patients, rapid identification of bacterial species may help to guide treatment at early stages. New protocols for the identification directly from positive blood culture flasks significantly helped in the presented case report.

**Case presentation:**

Two patients (a father and son) presented with diarrhea, malaise, and fever of 3 to 4 days duration. Blood cultures from both patients cultured short Gram-positive rods. MALDI-TOF based rapid identification protocol direct from positive blood culture identified *Listeria monocytogenes* as the cause of sepsis and could be confirmed with conventional methods. *Listeria monocytogenes* was identified 24 h later by conventional biochemical identification methods (VITEK 2). Antibiotic treatment was adjusted early in response to the MALDI-TOF based identification of bacteremia. Pulsed field gel electrophoresis confirmed the suspected relatedness of the father’s and son’s isolates.

**Conclusions:**

MALDI-TOF based may provide a rapid identification of bacterial species directly from positive blood culture.

## Background

*Listeria monocytogenes* is a rare but emerging foodborne pathogen with transmission predominantly by unprocessed contaminated food products [1]. Although outbreaks have been described, most reported cases remain sporadic [2]. In Switzerland, 43 and 96 cases have been reported in 2013 and 2014 respectively [3]. There is a wide spectrum of clinical manifestations with febrile gastroenteritis predominating, which is generally associated with a complete recovery. Invasive infections such as bacteremia, sepsis or meningoencephalitis can occur and are associated with a mortality rate approaching 20% [4, 5]. Therefore, rapid and reliable identification of the organism followed by institution of targeted antibiotic treatment is crucial. The rapid analysis of positive blood cultures using the SepsiTyper Kit may allow accurate and timely diagnosis approximately 24 h earlier than conventional microbiological approaches [6]. This rapid identification of the bacterial species and the subsequent adjustment of the antibiotic treatment may offer a substantial advantage in the treatment of patients with sepsis. Indeed, previous studies have shown a beneficial impact of MALDI-TOF based identification compared to gram staining and subsequent conventional biochemistry-based work-up. Using MALDI-TOF based identification directly on blood cultures significantly increased the rate of early antibiotic modification [7, 8]. Rapid identification of bacteria in patients with blood stream infection has been shown to improve the patients’ outcome by optimizing antibiotic treatment. Differences in mortality were only observed in combination with an antibiotic stewardship program [9]. We have recently performed a randomized trial to compare conventional identification versus Sepsityper based identification with MALDI-TOF. We could show that a more rapid optimization in antibiotic treatment resulted in a reduced mortality rate and admission rate to the intensive care unit [10]. In any case the identification of bacterial species from blood cultures should be confirmed with conventional culture based methods, as MALDI-TOF based identification cannot recognize mixed infections.

The aim of this case report is to discuss the unusual case of two patients both admitted to an intensive care unit due to blood stream infection with *Listeria monocytogenes*. We discuss the rapid identification process by using MALDI-TOF and discuss the literature of MALDI-TOF based identification of blood stream infections.

## Case presentation

### Case 1 (the son)

A 16-year old boy presented to the pediatric emergency department with a 1 week history of rhinitis and general malaise and reduced appetite, and mild watery diarrhea with abdominal pain for the last 4 days. On the day of admission he had developed fever, vomited once, and complained of bilateral lower limb pain. His father was also ill with fever and diarrhea. Both had been at a camping site in Germany 1 week previously. A third person (grandfather) did not fall ill.

The boy is generally in good health. He is known to have alpha thalassemia (heterozygote) and glucose-6-phosphate dehydrogenase deficiency (G6PD). He suffered from malaria in May 2006 and August 2014 after vacations in Kenya, his country of origin. Currently, he did not take any regular medication. There was no significant travel history in the preceding 6 months.

On presentation he was unwell, core body temperature measured 39 °C. His heart rate was 111/beats per min, his blood pressure was 124/63 mmHg. He was tender in his right and left iliac fossae with rebound tenderness noted on the right. The remaining physical examination was unremarkable apart from bilateral tender thighs.

Peripheral blood white cell count (WCC) was 8.65 × 10^9^/l, C-reactive protein (CRP) was 34 mg/l (<10 mg/l) and Procalcitonin level was 0.929 ng/ml, (<0.1 ng/ml). Liver and renal parameters were unremarkable. Creatinine kinase was 272 U/l (<270 U/l). Blood cultures were taken and he was admitted to the ward for intravenous rehydration with a presumed diagnosis of viral gastroenteritis. One day after admission, he remained unwell with high fever and crampy abdominal pain. Appendicitis was considered but this was ruled out following surgical assessment. A second set of blood cultures was taken. His CRP increased to 175 mg/l and WCC decreased (min. 3.08 × 10^9^/l). On day 3 of admission, the second set of blood cultures yielded Gram-positive rods. A rapid identification using MALDI-TOF mass spectrometry directly from the positive blood culture vials revealed *Listeria monocytogenes* (time to positivity 20 h and 36 min.; see microbiology results below for details). Further diagnostic tests (stool cultures for *Shigella*, *Salmonella* and *Campylobacter* and PCR for Adeno- and Rotavirus) were all negative. Due to the MALDI-TOF results, treatment with Amoxicillin i.v (25 mg/kg qid) and Amikacin (15 mg/kg qd) was initiated and the patient recovered rapidly within three days without any sequelae. Amoxicillin i.v. was continued for a total of 10 days and Amikacin was discontinued after 5 days.

### Case 2 (the father)

The day following the son’s admission to hospital, his 46-year old father presented to the emergency room with a 3-day history of heavy watery diarrhea (10–20 times a day), vomiting, weakness, joint and muscle pain, and distinct headache. On one occasion his temperature measured >38 °C. Previously he was healthy, did not take regular medication, and drank no alcohol.

At presentation, the patient was febrile (core body temperature 39.8 °C) with a heart rate of 95 bpm and blood pressure reading 100/65 mmHg. Clinical examination revealed bilateral conjunctivitis and hyperactive bowel sounds. Laboratory analysis showed a left-shift of neutrophils (65%, norm 5–15%), an elevated CRP of 214 mg/l (<10 mg/L) and a creatinine level of 117 μmol/l (range 49–97 μmol/l).

As he had travelled to Kenya a few months previously, malaria was excluded. After drawing blood cultures, antibiotic therapy with ceftriaxone 2 g IV per day and metronidazole 500 mg PO three times a day was commenced as empiric therapy for sepsis due to infectious diarrhea.

The following day a blood culture flagged positive and Gram staining revealed Gram-positive rods. Again, the rapid identification directly from the positive blood culture vials using MALDI-TOF mass spectrometry showed *Listeria monocytogenes* (see microbiology results below for details). Further diagnostic tests (stool cultures for *Shigella*, *Salmonella* and *Campylobacter* and *C.difficile*- and Norovirus-PCR) were negative. According to the MALDI-TOF findings, the antibiotic therapy was changed to amoxicillin 2 g every 4 h IV and gentamicin 80 mg every 8 h IV (1 mg/kg tid). The latter was stopped after 5 days as the patient’s condition improved. Meningitis was ruled out following a normal cerebrospinal fluid analysis. On day nine, the diarrhea significantly improved and the patient was discharged home in good condition with oral sulfamethoxazole/trimethoprim 800/160 mg 2 tablets every 8 h (5 mg/kg trimethoprim component tid) to complete a 14 day course of antibiotic treatment.

Further detailed history revealed that both, father and son, had eaten suspect not-well tasting cheese and ham sandwiches at the camping site about a few days before presentation. No one else in the family and, as far as we know, nobody else at the camping site developed illness. No other unusual food was eaten. Unfortunately, the sandwiches as the suspected source of infection could not be retrieved retrospectively.

### Microbiological work-up

#### Bacterial species identification

Classical Gram staining of positive blood cultures showed short Gram-positive rods. We then used the SepsiTyper Kit and matrix-assisted laser desorption/ionization time-of-flight (MALDI-TOF; Microflex, Bruker) mass spectrometry directly from positive blood cultures (BacT/ALERT 3D, FN/FA vials; bioMérieux) for species identification as previously described [10, 11]. The bacterial species identification could be performed directly from all positive blood cultures within 45 min. From blood culture samples, erythrocytes were lysed, and raisins to absorb antibiotics and cellular debris were filtered using the SepsiTyper Kit (Bruker) including a full protein extraction. Briefly, 200 uL of lysis buffer was added to 1000 uL of blood culture fluid and briefly vortexed. Afterwards, 800 uL of the solution was centrifuged with a specific inlay filter system (SigmaPrep spin columns, SC1000-1KT) to remove the raisins and cellular debris (2 min for 2000 g). Next, the supernatant was centrifuged to for a bacterial pellet (2 min for 13,000 g). Next, the pellet was washed once with 1 mL a specific washing buffer (SepsiTyper), followed by additional washing step with 300 uL deionized water and 700 uL of ethanol. After centrifugation (2 min, 15,000 g), the ethanol was removed and the pellet was dried. Finally, formic acid (10–20 uL) was added to dissolve the pellet (depending on the pellet size), the same volume of acetonitrile was added and briefly vortexed. After a final centrifugation step (2 min for 13,000 g) the supernatant was transferred onto the MALDI-TOF target plate. One microliter of matrix solution was added (Bruker). The raw spectra were processed using the MALDI Biotyper 3.1 software and database (Bruker) at default settings for identification of bacterial species.

Both blood cultures resulted in good MALDI-TOF scores of 2.332 and 2.124 respectively. We could confirm the species identification with conventional methods. Bacterial colonies were harvested and used for identification with biochemical reactions using the VITEK 2 system (bioMérieux). Classical identification was available 24 h later.

#### Typing of Listeria monocytogenes

**Fig. 1:**
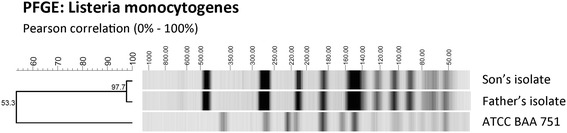
Pulsed field gel electrophoresis of *Listeria monocytogenes* isolates

We could prove the relatedness of both isolates using pulsed field gel electrophoresis (PFGE) as previously published [12, 13]. Our standard protocol was adapted slightly. For optimization of the running conditions, we used a series of bacterial concentrations and tested two restriction enzymes (*Apa I* and *Asc I*, data not shown). After DNA fragmentation with *Apa I*, we used a switch time of 4–40 as running specifications of the gel [14]. Afterwards, dendrograms were drawn with the use of GelCompar software (version 4.5, Applied Maths, Belgium). Figure 1 illustrates the close relatedness of the two isolates in comparison to an ATCC reference strain (Fig. 1).

## Discussion and conclusion

Invasive infections with *L. monocytogenes* (i.e., bacteremia, meningoencephalitis and endocarditis) mostly occur in immunocompromised or elderly patients [15]. Underlying immunocompromising diseases include conditions such as malignancies, diabetes mellitus, liver disease and alcoholism. Immunosuppressive treatments comprise glucocorticoid therapy and TNF-alpha antagonists, and many others [16]. Another well-known predisposing condition is pregnancy with the associated risk of transplacental transmission. Only one-third of listeriosis occurs in persons without a predisposition [5]. In our cases, we could not identify an immunodeficiency or predisposing factor in either patient. Although, the son’s diagnosis of alpha thalassemia could represent a potential risk factor, a literature search did not reveal any known associations. We seek to highlight the very unusual presentation of two young and otherwise healthy, immunocompetent individuals falling seriously ill with bacteremia due to *L. monocytogenes*.

There are no explicit guidelines for the treatment of listeria infections. In general, febrile gastroenteritis does not require antimicrobial therapy. In selected patients such as the immunocompromised or pregnant women, oral ampicillin or trimethoprim-sulfamethoxazole for several days is recommended, especially if they suffer protracted symptoms [17]. Cephalosporines demonstrate no activity against *L. monocytogenes*. For invasive *Listeria* infections, high dose ampicillin intravenously is the treatment of choice [18, 19]. Combination therapy with an aminoglycoside should be considered to treat listerial central nervous system infections, endocarditis, and infections in neonates and immunocompromised patients [16, 20]. The optimal duration of therapy is unknown and may be influenced by the site of infection [21, 22]. Extended courses are recommended in immunocompromised patients and those with CNS infections [16, 19]. In our patients, a 10 to 14 day course of antimicrobial therapy was deemed sufficient. Follow-up at 2 weeks confirmed the complete recovery of both patients without sequelae.

In summary, these two epidemiologically linked cases of invasive *Listeria monocytogenes* infection demonstrate the usefulness of MALDI-TOF mass spectrometry based rapid identification to a species level directly from positive blood cultures. MALDI-TOF may provide a timely identification of unusual, more rare pathogens and institution of the required specific therapy. However, MALDI-TOF results directly from blood culture should be confirmed with conventional culture based methods to recognize mixed infections.

## Abbreviations

CRP: C-reactive protein
G6PD: Glucose-6-phosphate dehydrogenase deficiency
MALDI-TOF: Matrix assisted laser desorption ionization – time of flight
WCC: White cell count

## References

[1] Schlech WF (2000). Foodborne listeriosis. Clin Infect Dis.

[2] Varma JK, Samuel MC, Marcus R, Hoekstra RM, Medus C (2007). Listeria monocytogenes infection from foods prepared in a commercial establishment: a case–control study of potential sources of sporadic illness in the United States. Clin Infect Dis.

[3] Gesundheit BF. Wöchentliche Fallzahlen, Meldungen Infektionskrankheiten. BAG Bulletin; 2015

[4] Scallan E, Hoekstra RM, Angulo FJ, Tauxe RV, Widdowson MA (2011). Foodborne illness acquired in the United States–major pathogens. Emerg Infect Dis.

[5] Gesundheit Bf (2001). Die Listeriose in der Schweiz - Empfehlungen zu Prävention, Diagnose und Therapie. BAG Bulletin.

[6] Dierig A, Frei R, Egli A (2015). The fast route to microbe identification: matrix assisted laser desorption/ionization-time of flight mass spectrometry (MALDI-TOF MS). Pediatr Infect Dis J.

[7] Prod’hom G, Bizzini A, Durussel C, Bille J, Greub G (2010). Matrix-assisted laser desorption ionization-time of flight mass spectrometry for direct bacterial identification from positive blood culture pellets. J Clin Microbiol.

[8] Clerc O, Prod’hom G, Vogne C, Bizzini A, Calandra T (2013). Impact of matrix-assisted laser desorption ionization time-of-flight mass spectrometry on the clinical management of patients with Gram-negative bacteremia: a prospective observational study. Clin Infect Dis.

[9] Vardakas KZ, Anifantaki FI, Trigkidis KK, Falagas ME (2015). Rapid molecular diagnostic tests in patients with bacteremia: evaluation of their impact on decision making and clinical outcomes. Eur J Clin Microbiol Infect Dis.

[10] Osthoff M, Gürtler N, Bassetti S, Balestra G, Marsch S, Pargger H, Weisser M, Egli A. Impact of MALDI-TOF-MS-based identification directly from positive blood cultures on patient management: a controlled clinical trial. Clin Microbiol Infect. 2016. doi:10.1016/j.cmi.2016.08.009. 10.1016/j.cmi.2016.08.009 27569710

[11] Egli A, Osthoff M, Goldenberger D, Halter J, Schaub S (2015). Matrix-assisted laser desorption/ionization time-of-flight mass spectrometry (MALDI-TOF) directly from positive blood culture flasks allows rapid identification of bloodstream infections in immunosuppressed hosts. Transpl Infect Dis.

[12] Egli A, Tschudin-Sutter S, Oberle M, Goldenberger D, Frei R (2015). Matrix-assisted laser desorption/ionization time of flight mass-spectrometry (MALDI-TOF MS) based typing of extended-spectrum beta-lactamase producing E. coli–a novel tool for real-time outbreak investigation. PLoS One.

[13] Stranden A, Frei R, Widmer AF (2003). Molecular typing of methicillin-resistant Staphylococcus aureus: can PCR replace pulsed-field gel electrophoresis?. J Clin Microbiol.

[14] Luque-Sastre L, Fanning S, Fox EM (2015). Pulsed-field gel electrophoresis for Listeria monocytogenes. Methods Mol Biol.

[15] Bula CJ, Bille J, Glauser MP (1995). An epidemic of food-borne listeriosis in western Switzerland: description of 57 cases involving adults. Clin Infect Dis.

[16] Mylonakis E, Hohmann EL, Calderwood SB (1998). Central nervous system infection with Listeria monocytogenes. 33 years’ experience at a general hospital and review of 776 episodes from the literature. Medicine (Baltimore).

[17] Ooi ST, Lorber B (2005). Gastroenteritis due to Listeria monocytogenes. Clin Infect Dis.

[18] Lorber B (1997). Listeriosis. Clin Infect Dis.

[19] Lorber B, Mandell GL, Bennett JE, Dolin R (2010). Listeria monocytogenes. Principles and practice of infectious diseases.

[20] Hof H, Nichterlein T, Kretschmar M (1997). Management of listeriosis. Clin Microbiol Rev.

[21] Merle-Melet M, Dossou-Gbete L, Maurer P, Meyer P, Lozniewski A (1996). Is amoxicillin-cotrimoxazole the most appropriate antibiotic regimen for listeria meningoencephalitis? Review of 22 cases and the literature. J Infect.

[22] Spitzer PG, Hammer SM, Karchmer AW (1986). Treatment of Listeria monocytogenes infection with trimethoprim-sulfamethoxazole: case report and review of the literature. Rev Infect Dis.

